# Advances of single-cell genomics and epigenomics in human disease: where are we now?

**DOI:** 10.1007/s00335-020-09834-4

**Published:** 2020-04-08

**Authors:** Rizqah Kamies, Celia P. Martinez-Jimenez

**Affiliations:** Helmholtz Pioneer Campus, Helmholtz Zentrum München, 85764 Neuherberg, Germany

## Abstract

Cellular heterogeneity is revolutionizing the way to study, monitor and dissect complex diseases. This has been possible with the technological and computational advances associated to single-cell genomics and epigenomics. Deeper understanding of cell-to-cell variation and its impact on tissue function will open new avenues for early disease detection, accurate diagnosis and personalized treatments, all together leading to the next generation of health care. This review focuses on the recent discoveries that single-cell genomics and epigenomics have facilitated in the context of human health. It highlights the potential of single-cell omics to further advance the development of personalized treatments and precision medicine in cancer, diabetes and chronic age-related diseases. The promise of single-cell technologies to generate new insights about the differences in function between individual cells is just emerging, and it is paving the way for identifying biomarkers and novel therapeutic targets to tackle age, complex diseases and understand the effect of life style interventions and environmental factors.

## Introduction

Recently, efforts have been made to highlight the importance of moving translational genomic findings to the clinic for the overall improvement of human health (Cho et al. 2016; Regev et al. 2017; Zeggini et al. 2019). Accordingly, these would include the translation of, but are not limited to the experimental discovery of results, the analysis and functional interpretation of results, the generation of large-scale data and the utilization of advanced computational software to handle result output and lastly, the application of result findings in a clinical setting (Behjati et al. 2018; Haghverdi et al. 2016; Zeggini et al. 2019). These applications, in combination with the approval of multiple other ethical, legal, social, economic and political factors could be used to ultimately combat disease, detect early onsets of disease, monitor disease progression and potentially facilitate preventative treatments (Behjati et al. 2018; Gomes et al. 2019; Regev et al. 2017; Zeggini et al. 2019). Although this approach has been successfully applied in some monogenic disorders and in rare disease cases where precision medicine techniques are used as a specific or preventative treatment (June et al. 2018; Karczewski and Snyder 2018; Zeggini et al. 2019), the implementation of this comprehensive translational genomics approach to complex chronic diseases in humans is yet to be achieved (Grouse 2015; Regev et al. 2017).

While the analysis of multiple “omic” (genomic, transcriptomic, proteomic and metabolomic) molecular profiles in bulk have been well established to study cellular homeostasis and disruptions as a consequence of disease (Hasin et al. 2017; Karczewski and Snyder 2018; Sun and Hu 2016), most genetic and epigenetic mechanisms are yet to be probed with single-cell resolution. To understand the finer details at the level of a singular cell, sophisticated genomic and epigenomic next-generation sequencing (NGS) technologies have increased the potential for research output immensely (see Clark et al. 2018; Clark et al. 2016; Kelsey et al. 2017; Macaulay et al. 2017; Stuart and Satija 2019). These would include whole-genome profiling techniques of RNA, DNA, proteins, epigenetic modifications, chromatin accessibility and chromosome conformations on the level of an individual cell (described in Clark et al. 2016; Kelsey et al. 2017; Macaulay et al. 2017; Mincarelli et al. 2018; Nagano et al. 2017; Svensson et al. 2018; Wagner et al. 2016). In this review, we will provide a concise description of the impact of single-cell technologies in the context of human health and disease, while technical development and computational analysis required for the near-future translational applications of the single-cell genomic discoveries are reviewed elsewhere (see Birnbaum 2018; Luecken and Theis 2019; Song et al. 2019; Tang et al. 2019; Wang and Song 2017). The harmonization and standardization of single-cell technologies will lead to unprecedented discoveries and translational applications from bench to bed (Shalek and Benson 2017; Strzelecka et al. 2018; Wang and Song 2017).

## The individuality of cells

Epigenetic programs are decisive for cell fate decisions, cell identity and cell state (Borsos and Torres-Padilla 2016; Fischer et al. 2019; Trapnell 2015). When RNA transcripts and components of the epitranscriptome initiate a cascade of events in cells, in response to extrinsic or intrinsic stimuli, single-cell genomics and epigenomics can be used to effectively quantify and monitor those dynamic or discrete changes (Clark et al. 2018; Goldman et al. 2019; Mincarelli et al. 2018; Tritschler et al. 2019). This approach is especially important in a seemingly homogenous population of cells, where in most cases, cells are isolated from the same tissue and epigenomic signatures underlying disease are often concealed in bulk samples (Kelsey et al. 2017; Strzelecka et al. 2018; Tritschler et al. 2019; Wang et al. 2018). Additionally, distinguishing the precise intercellular differences is challenging when considering thousands of cells simultaneously. Often, only the most frequent or the most abundant molecular feature is the one detected on average in a given cell population. (Goldman et al. 2019; Haghverdi et al. 2016; Trapnell 2015). Although cellular heterogeneity is essential to the survival of a population, where increased diversity in cells allows increased adaptation to changes in the surrounding milieu (Goldman et al. 2019), increases in cell-to-cell variability have also been associated to age and age-related diseases (Enge et al. 2017; Hernando-Herraez et al. 2019; Martinez-Jimenez et al. 2017). Moreover, a deep understanding of cellular variability and the impact of this variability in tissue function will allow us to understand how changes in cellular dynamics can influence the entire organism and even lead to cancer, diabetes, metabolic disorders and accelerated ageing (Cheung et al. 2018; Ecker et al. 2018; Enge et al. 2017; Hurria et al. 2016).

**Fig. 1:**
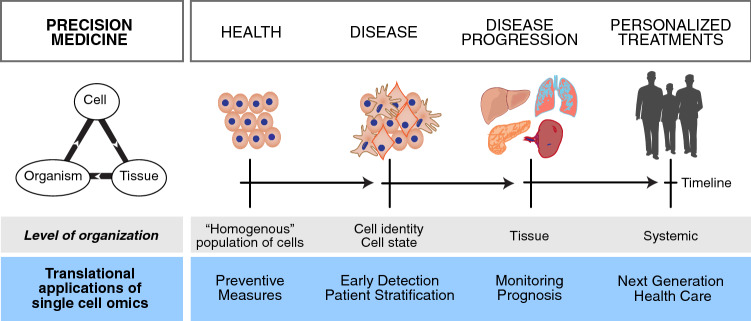
Single-cell omics is advancing the development of personalized treatments. Precision medicine utilizes genetic information from all levels of cellular organization (cell, tissue, organism) obtained from patient data, to tailor treatments. These novel technologies investigate how cells from a healthy, seemingly homogenous population of cells can lead to a population with different cellular states, triggering tissue dysfunction and systemic effects (highlighted in grey). The translational applications of single-cell omics will impact on preventive measures, early detection and disease monitoring, leading to the next generation of health care (highlighted in blue)

To effectively capture and observe the morphological and phenotypical differences of cells in a healthy and diseased state using single-cell approaches, the concept of ‘cell identity’ should be more concisely understood (see Fig. 1). Although no standardized method for defining ‘cell identity’ exists (Morris 2019), elucidation on these definitions can be given to make accurate functional-based assumptions on what changes a cell might undergo until it reaches its final destination or ‘cell fate’ (Kelsey et al. 2017; Morris 2019; Trapnell 2015; Tritschler et al. 2019). Briefly, cell identity can be deconvolved into a ‘cell type’ and a ‘cell state’ within a spatiotemporal manner (Camp et al. 2019; Haghverdi et al. 2016; Treutlein et al. 2014). A cell type or sub-type refers to an observable, functional change within a population where properties vary distinctly in response to extrinsic factors, while a cell state refers to a dynamic change that alters the phenotype and function of the cell in a continuous manner and is often regulated intrinsically (see Camp et al. 2019; Chen et al. 2018; Mincarelli et al. 2018; Morris 2019; Trapnell 2015). For instance, the hematopoietic stem cell (HSC) is a well-elaborated example, where a cell type has the ability to undergo various functional states in order to achieve mature blood and immune cell proliferation after transplantation in a continuous manner, through its multi-lineage potential and the ability to self-renew (Becker et al. 1963; Mincarelli et al. 2018; Velten et al. 2017). In a nutshell, within the context of health and disease, if we know where a cell comes from, its inherent function and how these functions and states change accordingly to various stimuli, we could predict its fate, with the goal of anticipating the development of a disease or monitoring its progression and outcome (Fig. 1).

These thoughts are echoed by a consortium of researchers who have come together to generate a Human Cell Atlas (Regev et al. 2017), which is an initiative to identify and map all the different cell types in the human body (much like an atlas) for referral of healthy and diseased cells, tissues, organs and systems (Regev et al. 2017; Rozenblatt-Rosen et al. 2017; Strzelecka et al. 2018). The project aims to have a comprehensive composition of all the cell types and identities in the human body, allowing the identification of patterns and interactions at various levels of resolution and to facilitate a functional starting point for researchers when trying to answer relevant health-related questions (Regev et al. 2017; Rozenblatt-Rosen et al. 2017). Subsequently, the next step moving forward with a fully annotated and functional human cell atlas would be to concentrate on reducing disease damages, develop methods of preventative treatment, improve disease diagnostics and advance personalized medicine (Strzelecka et al. 2018). Accordingly, these goals are aligned with those of the LifeTime Initiative (https://lifetime-fetflagship.eu/), a unified research initiative to understand the cause and biological mechanisms behind disease, monitor and track disease changes and progression and ultimately, treat individual human cells affected by disease.

Thus far, such progressive atlas initiatives have been achieved in the model organism *Mus musculus* through an initiative of the *Tabula Muris* Consortium (Tabula Muris et al. 2018), where information from a transcriptomic analysis of more than 100 000 cells collected from 20 organs and tissues was collated to establish a foundation for an atlas *in mouse* (Tabula Muris et al. 2018). In humans, the effect would be far more complex as each and every person is different and each and every organ or tissue is affected differently in diseased situations. Furthermore, for complex chronic diseases known to affect human health and expedite the ageing process, single-cell genomic and epigenomic techniques have become necessary for early disease detection, accurate diagnosis and prognosis, monitoring disease progression in tissues and systemically, to facilitate personalized treatment and achieve next-generation health care (see Fig. 1). Here we review recent key findings.

## The influence of single-cell approaches

### Cancer

To date, tumour-biology studies have represented one of the biggest challenges in improving targeted cancer therapies (Levitin et al. 2018; Strzelecka et al. 2018). Moreover, because tumours are an elaborate, complex mix of different cell types and states arising from a single cell that has progressed and diversified through somatic mutations to form distinct subpopulations (Qian et al. 2017; Sierant and Choi 2018; Suvà and Tirosh 2019), intercellular heterogeneity is critical for both accurate diagnosis and personalized treatment (Qian et al. 2017; Sant et al. 2017). The accurate profiling of cellular variability at the single-cell level within a tumour environment where both malignant and immune cells are present (amongst others) could locate the correct gene sequences (biomarkers) for targeted treatment and improve treatment efficiency, specifically through administration of the appropriate drugs to prevent or reduce cancer relapse (Qian et al. 2017; Sierant and Choi 2018; Wagner et al. 2019). This is especially important when only a specific part of the tumour is targeted and proliferation is still possible even from a minute proportion of cells, often concealed in bulk analyses (Qian et al. 2017; Tirosh and Suvà 2019). For example, in studies investigating lung adenocarcinomas, accurate profiling of tumours for targeted treatment remains challenging due to increased tumour heterogeneity (Zhang et al. 2019). While mutations of the epidermal growth factor receptor (*EFGR*) in non-small cell lung cancers (NSCLC) have been widely employed as biomarkers for lung carcinogenesis (Harrison et al. 2019; Zhang et al. 2019), resistance to the well-established EGFR tyrosine kinase inhibitors has been frequently reported, most likely due to *EGFR* T790M resistance mutations (Del Re et al. 2018; Rexer et al. 2009; Sullivan and Planchard 2016). Although classical histomorphologic evaluation of a malignant tumour is argued to be the most significant diagnostic, prognostic and predictive biomarker with the greatest impact on patient treatment (Radpour and Forouharkhou 2018), in-depth single-cell RNA sequencing in combination with protein profiling of primary human tumours has been proposed to be highly effective in discerning cell types and subtypes within tumour cells and for detection of distinct functional states of proliferation (Tirosh and Suvà 2019).

To elaborate, an integrated analysis of cancer cells has been shown in hepatocellular carcinoma (HCC) where time-of-flight mass cytometry (CyTOF) and single-cell RNA sequencing were used to show an immunosuppressive gradient of immune cells in a tumour microenvironment, non-tumour microenvironment and peripheral blood with the goal of describing the phenotypical characteristics of the T-cell subsets (Chew et al. 2017). The authors describe the enrichment of regulatory T-cells, CD8+ T-cells and natural killer cells in the tumour microenvironment in conjunction with the expression of multiple markers for T-cell exhaustion, when compared to T-cell subsets in the non-tumour microenvironment (Chew et al. 2017). In high-grade serous ovarian cancer (HGSOC), CyTOF was employed with un-supervised computational analysis for an in-depth phenotypical characterization of both dominant and rare cell phenotypes linked to surface markers, intracellular signalling proteins, transcription factors and cell-cycle proteins active in the malignancy (Gonzalez et al. 2018). Recently, a pilot study involving human bone marrow cells has been subjected to combinatorial single-cell RNA sequencing, multiparameter flow cytometry and mass cytometry to show diversity across human samples over the full range of adulthood and to act as a reference for different cell populations (Oetjen et al. 2018).

For further investigation of solid tumours, the invasive human epidermal growth factor 2 (HER2) protein part of the ERBB pathway is active in numerous cancers including pancreatic, ovarian, breast, gastric, lung, glioma/glioblastoma, colorectal, in the central nervous system, and is one of the most well-studied oncogenes (Masoud and Pagès 2017; Townsend et al. 2018). In particular, the cancer-related *HER2* mutation is often used as a biomarker for 15–20% of all breast cancer tumours (Rye et al. 2018) and has been successfully targeted for gene therapy (Chung et al. 2017; Masoud and Pagès 2017). Moreover, the oncogene has been extensively studied with single-cell RNA sequencing, particularly to show extensive intratumoral heterogeneity (Cho 2019; Chung et al. 2017; Wang et al. 2019a, b). Recently, Cho (2019) showed triple-negative breast cell populations identified by three subtyping marker genes (*ERBB2* also known as *HER2, ESR1* and *PG)*, while immune landscape cell populations, consisting of subclasses of both tumour and non-tumour (immune) cells, were shaped by distinctive gene expression signatures inferred from copy number alterations within the tumour microenvironment by Chung et al. (2017). Subsequent single-cell RNA sequencing investigations of *HER2*+ with the monoclonal antibody, Trastuzumab (Herceptin), successful in targeting HER2+ breast cancer cells (Wang et al. 2019b), re-affirmed previously highlighted gene sequences such as *CLU* and *SEPP1* genes in trastuzumab-treated patients and indicated new gene signatures of interest such as the chemokine ligands *CXCL1* and *CXCL8* that were significantly downregulated under trastuzumab treatment (Wang et al. 2019a). In addition, further single-cell RNA sequencing of trastuzumab resistance patients has also been performed (Wang et al. 2019b), where studies have shown that perhaps a combination of inhibitors targeting CDK4/6 inhibitor-resistant tumours are required, specifically those targeting a immunosuppressive immature myeloid cell (IMC) population in resistant tumours (Wang et al. 2019b).

The *KRAS* mutation has been reported to be the most frequently mutated oncogene in human tumours and has been the investigative target in many cancer studies specifically in colorectal, pancreatic and in non-small lung cancers (Kim et al. 2018; Kuboki et al. 2019; Roerink et al. 2018; Román et al. 2018). The extensive mutation rate of the *KRAS* oncogene has played detrimental roles in cancer initiation, propagation and maintenance and thus could be highlighted as a therapeutic target for specific treatment (Cox et al. 2014; Kim et al. 2018). Recently, the CRISPR-Cas9 system has been employed as a proof of concept study, where guide RNAs specific to targeted gene sequences present on mutant *KRAS* alleles were employed with the goal of removing gene sequences known to cause malignancies (Kim et al. 2018). Although, the study proved successful in targeting mutant gene sequences for manipulation, the authors conclude that the technique alone is not enough to induce tumour remission, but could be considered as a gene therapy to reduce tumour volume by blocking tumour growth in vivo before surgery (Kim et al. 2018).

The literature reported on targeted cancer biomarkers thus far is considerable, many of which include Chimeric Antigen Receptor (CAR) T-cell therapy that have been successful or have been making progress in personalized cancer treatment (see Townsend et al. 2018). These genetically engineered CAR T-cells have shown immunotherapy action against numerous haematological malignancies, some of which include the famous CD19 protein for treatment of acute lymphoblastic leukaemia and large B-cell lymphoma (Feins et al. 2019) and new receptor targets (CD5, CD123, CD33, CD70, CD38, and BCMA) that are currently being evaluated and have already shown positive results (Townsend et al. 2018).

## Diabetes

The main source of diabetes development and disease progression has been attributed to disturbances in the regulation and synchronization of the hormone-producing cells in the pancreatic islets (Carrano et al. 2017). These cells are clusters of at least five different endocrine cell types (alpha, beta, delta, gamma and epsilon) that each produce a unique hormone to function together in a well-orchestrated manner in controlling and maintaining blood glucose levels (Da Silva Xavier 2018). While alpha and beta endocrine cells are stimulated to release glucagon and insulin, respectively (Da Silva Xavier 2018; Theis and Lickert 2019), discordance in both cell types has been shown to increase disease pathogenesis in type 1 and type 2 diabetes (Ackermann et al. 2016; Brissova et al. 2018; Tritschler et al. 2017). Moreover, an increase in hyperglycaemia has been associated with a loss of beta-cell mass, function and organization and is the cell type most frequently studied for insulin resistance (Carrano et al. 2017; Lawlor et al. 2017b; Segerstolpe et al. 2016; Theis and Lickert 2019; Tritschler et al. 2017).

Notably, single-cell transcriptome profiling has been utilized in the past few years to discern cellular heterogeneity within the islets of Langerhans (Fischer et al. 2019; Tritschler et al. 2019, 2017), particularly for beta cells (Baron et al. 2016; Lawlor et al. 2017a; Segerstolpe et al. 2016; Teo et al. 2018; Xin et al. 2016). Segerstolpe et al. (2016) investigated cell-type specific gene expression in the pancreas of healthy and type 2 diabetic individuals and uncovered major gene expression differences (transcriptional signatures) between exocrine and endocrine cell types, including the less abundant cell types such as human delta, gamma and epsilon cells. Previously, these cells had been difficult to observe due to bulk characterization methods (Lawlor et al. 2017a), however, single-cell RNA sequencing has shed light on the novel roles for each rare cell type based on their activated signalling pathways and receptor proteins (Lawlor et al. 2017a; Segerstolpe et al. 2016). For example, insight into the transcriptome of the minority cell type, epsilon cells and its ghrelin-producing capability was provided (Segerstolpe et al. 2016), as well as the expression of the rare delta and gamma cell types that are prompted by hormonal cues from leptin, ghrelin and dopamine signalling pathways to facilitate metabolic signalling in the pancreas (Lawlor et al. 2017a). Further single-cell RNA investigations by Xin et al. (2016) showed a total of 245 genes to be affected by type 2 diabetes when compared to non-diabetic single-cell transcriptomes. Among the common transcript expression profiles found between the human islet cells, only 20 genes (for example, *RBP4, DLK1, ADCYAP1, RGS16, SOX4*, *BMP5, TIMP2, TSPAN1, MAFB* and *TFF3*) were specific to a certain cell type (Xin et al. 2016). Lastly, a few recent reviews have tracked the progress of genes linked to specific endocrine cell types in these studies (see Chiou et al. 2019; Tritschler et al. 2017), with some going as far as to re-analyse the single-cell transcriptome datasets using a machine learning approach (Ma and Zheng 2018). The in-depth analyses reported on oxidative stress being the perpetrator to enhance beta-cell dysfunction as a final result, together with the potential activation of pathways linked to beta-cell apoptosis that may be the resulting cause of an insulin gene expression deficit in type 2 diabetes (Ma and Zheng 2018).

Furthermore, there has been a notion that alpha cells have the ability to transdifferentiate into beta cells under conditions of extreme metabolic tress or when prompted under strong metabolic signalling (Ackermann et al. 2016; Tritschler et al. 2017). This has been postulated to be in part, due to the flexibility in the epigenome of alpha cells (Ackermann et al. 2016), where multiple bivalent activating and repressing histone marks (H3K4me3 and H3K27me, respectively) on gene loci associated with alpha and beta cells have been identified (Ackermann et al. 2016; Bramswig et al. 2013). In addition, more areas of open chromatin (~ 75%) in alpha cells, in comparison to beta cells have been detected, many of which were associated with beta-cell signature genes (Ackermann et al. 2016; Tritschler et al. 2017). Recently, efforts have been made to observe cell-type specific transcriptomes mapped to areas of open chromatin to define gene regulatory regions, characterize novel gene signatures and highlight transcription factors of interest pertaining to diabetes pathogenesis (Ackermann et al. 2016; Bysani et al. 2019; Chiou et al. 2019; Rai et al. 2019). Most of these studies have used the Assay for Transposase Accessible Chromatin Sequencing (ATAC-seq) technique (Buenrostro et al. 2015; Lareau et al. 2019) for profiling rare and common endocrine cell types.

An intriguing study by Chiou et al. (2019) used a sophisticated new approach to obtain ATAC-seq profiles from single nuclei (snATAC-seq) to show differentiated regions of open chromatin from heterogenous cell types and subtypes with the aim of highlighting molecular mechanisms linked to genetic risk variants of type 2 diabetes. The authors were able to localize 239 fine-mapped type 2 diabetes risk signals to areas of open chromatin and ordered variants in islets at these signals with predicted regulatory functions to known target genes such as the *KCNQ1* locus (Chiou et al. 2019). Further insight into how type 2 diabetes alters chromatin organization and allows the subsequent affinity for suitable transcription factors and thus gene expression in pancreatic islets was provided by Bysani et al. (2019). A total of 1078 regions of open chromatin corresponding to 898 genes were detected and differentially expressed between diabetic and non-diabetic islets, many of which were annotated to genes linked to islet dysfunction and type 2 diabetes instigators such as *HHEX, HMGA2, GLIS3, MTNR1B*, *PARK2* and some previously associated single-nucleotide polymorphisms (SNPs) (Bysani et al. 2019). Furthermore, a large proportion of ATAC-seq peaks were mapped near to transcription start sites for easy manipulation by cis-regulatory elements, particularly enhancers and in areas where cell-type specific transcription factors such as FOXA, MAFB, NKX2.2, NKX6.1 and PDX1 for type 2 diabetes bind (Bysani et al. 2019). It is important to note that the overall goal of observing cell-type expression profiles contributing to type 2 diabetes is to reveal novel druggable targets for pathway manipulation and further approaches to prevent, monitor and treat type 2 diabetes (Chiou et al. 2019; Lawlor et al. 2017a; Tritschler et al. 2017).

### Chronic and age-related diseases

Ageing can be defined as the progressive decline in physiological and cellular functions (Enge et al. 2017; López-Otín et al. 2013). Although ageing has not been classified as a disease by the World Health Organization (WHO), it is considered a leading risk factor for all chronic diseases (WHO 2009). From epidemiological studies and experimental data, we know that chronic diseases accelerate the ageing process, suggesting that ageing and chronic diseases share common molecular mechanisms (Kennedy et al. 2014). For instance, the incidence of cancer, diabetes, kidney disease (O’Sullivan et al. 2017; Rowland et al. 2018) and non-alcoholic fatty liver disease (NAFLD) (Hunt et al. 2019; Ogrodnik et al. 2017) increases with age. Age-related disease in the kidney has been linked to nephrosclerosis, impaired renal function and chronic kidney disease (O’Sullivan et al. 2017; Rowland et al. 2018). In the liver, NAFLD is the most common chronic liver disease and comprises a range of related disorders in which the earliest stage is the accumulation of lipids in the liver followed by steatohepatitis that is generally marked by liver inflammation. Later on, steatohepatitis may progress to liver fibrosis and liver failure. Non-alcoholic fatty liver and steatohepatitis are reversible in their early stages, but ultimately may lead to the development of cirrhosis (that is an irreversible state) and hepatocellular carcinoma (HCC) (Podrini et al. 2013).

Recently, single-cell RNA sequencing has been used to study the complex cellular architecture of the kidney and investigate how changes in gene expression patterns are associated with chronic kidney disease (Chen et al. 2017; Der et al. 2017; Lake et al. 2019; Liao et al. 2020; Park et al. 2019, 2018). The human kidney is a highly complex tissue comprised of at least 30 different cell types that function in an intricate filtration system to remove nitrogen, water and other waste products from the blood, maintain electrolyte balance and red blood cell production and regulate blood pressure through hormonal secretion (Lake et al. 2019; Park et al. 2018; Rowland et al. 2019). Park et al. (2018) profiled approximately 58,000 cells isolated from heathy mouse kidneys and discovered that 21 homologous genes in humans were associated with monogenic inheritance of proteinuria and other complex-trait diseases such as chronic kidney disease and nephrolithiasis (Park et al. 2018). Similarly, by means of single-nucleus RNA sequencing (snRNA-seq), Lake et al. (2019) have shown the power of this technology to analyse clinical samples bypassing technical limitations in the enzymatic dissociation of the solid tissue and using limiting amounts of sample (Lake et al. 2019). Further analysis of receptor-ligand signalling pathways among cell types showed how the dysregulation of expression profiles associated to integrins in multiple cell types play a major role in the development of human kidney disease (Lake et al. 2019).

Likewise, single-cell genomics has emphasized the cellular heterogeneity present in the liver, with regard to the liver zonation of hepatocytes (Aizarani et al. 2019; Dobie et al. 2019; Halpern et al. 2018, 2017; Ramachandran et al. 2019) and the variability among non-parenchymal cells during chronic liver disease (Krenkel et al. 2020; MacParland et al. 2018; Pepe-Mooney et al. 2019; Su et al. 2017; Xiong et al. 2019). MacParland et al. (2018) identified 20 distinct cell populations of hepatocytes, endothelial cells, cholangiocytes, hepatic stellate cells and resident cells from the immune compartment. Beyond a liver cell atlas, this work identified two different populations of intrahepatic CD68^+^ macrophage populations with inflammatory or immunoregulatory properties, respectively (MacParland et al. 2018). Later on, Aizarani et al. (2019) sequenced CD45^+^ and CD45^−^ cells isolated from hepatocellular carcinomas from three patients showing how the gene expression signatures and biomarkers of liver cell types can be monitored in human liver disease.

Other chronic liver diseases such as Non-Alcoholic SteatoHepatitis (NASH) and liver fibrosis have also been investigated at the single-cell level (Dobie et al. 2019; Ramachandran et al. 2019; Xiong et al. 2019). The intercellular signalling between non-parenchymal cells (endothelial cells, Kuppfer cells and cholangiocytes) was analysed with single-cell transcriptomics and secretome analysis to reveal intercellular cross-talk via ligand and receptor signalling, in diet-induced NASH mice livers (Xiong et al. 2019). Comparative studies between mouse and human revealed a highly conserved pattern among liver cell types across species (Xiong et al. 2019). In addition, a novel NASH-specific macrophage population (termed NAM) was identified, suggesting that NASH alters the functional properties of liver macrophages populations by increasing Trem2 protein levels in a subset of macrophages, therefore increasing liver heterogeneity during pathogenesis of NASH (Xiong et al. 2019).

Recently, more than 100,000 cells isolated from healthy and cirrhotic human livers were analysed by single-cell RNA sequencing to further characterize the fibrotic niche and the cross-talk between non-parenchymal cells in the liver (Ramachandran et al. 2019). Ramachandran et al. (2019) have identified a scar-associated TREM2^+^ CD9^+^ subpopulation of macrophages that expands in liver fibrosis with a pro-fibrogenic phenotype. Additional endothelial subpopulations characterized by a high expression of PLVAP, CD34 and ACKR1, and mesenchymal cells expressing PDGFRA were also expanded in the pathogenesis of the liver disease (Ramachandran et al. 2019). Moreover, the zonation pattern of hepatic stellate cells across the hepatic lobe was also altered during liver fibrosis (Dobie et al. 2019). In summary, the unbiased analysis of the multi-lineage interactome between healthy and disease-affected livers will uncover novel molecular pathways which are potentially druggable, leading us to a new era of precision medicine (Ramachandran et al. 2019).

## Single-cell genomics and organoids: emerging diagnostic tool to understand tissue development and human disease

As the scope for human biological research expands, new personalized in vitro models are emerging as a diagnostic tool to study disease development and progression. These three-dimensional tissue cultures, termed organoids, are initiated from either pluripotent embryonic stem cells, induced pluripotent stem cell counterparts or tissue-resident adult stem cells (Clevers 2016; Huch et al. 2017; Lancaster and Huch 2019). Organoids recapitulate with sufficient complexity, molecular and cellular processes present in the original tissue (Camp and Treutlein 2017; Lancaster and Huch 2019). Therefore, this powerful system replicates in vitro*,* some level of organ development and disease phenotypes for a wide variety of tissues (Camp et al. 2019, 2018; Clevers 2016; Huch et al. 2017). These attributes make organoid models an ideal system to study complex disease phenotypes and implement personalized therapies in a controlled environment (Camp et al. 2019; Camp and Treutlein 2017). In addition, human organoids are genetically stable, can be long-term expanded in vitro and are composed of a collection of differentiated cell states that mimic the cellular composition in the corresponding original organ (Grassi et al. 2019; Hu et al. 2018; Huch et al. 2017; Lancaster and Huch 2019). For these reasons, the combination of single-cell omics and organoids has become an exceptional in vitro tool to dissect the molecular mechanisms underlying complex human diseases (Camp et al. 2019; Camp and Treutlein 2017).

At present, single-cell genomics has been applied to organoids modelling several human tissues (Brazovskaja et al. 2019) including brain (Camp et al. 2015; Kanton et al. 2019; Tanaka et al. 2020), kidney (Harder et al. 2019), liver (Camp et al. 2017; Huch et al. 2015), lung (Lee et al. 2017; Sachs et al. 2019) and intestine (Mithal et al. 2020). This line of research has advanced our understanding of the molecular pathways involved in the pathogenesis of the disease, for instance in glomerular disease of the kidney (Harder et al. 2019), inflammatory bowel disease in the intestine (Mithal et al. 2020) and respiratory viral infection in the lung (Sachs et al. 2019). In particular, recent studies have shown the translational applications of liver organoids to model hepatic steatosis (Kruitwagen et al. 2017), steatohepatitis (Ouchi et al. 2019), alcohol liver injury (Wang et al. 2019c) and alpha-1 antitrypsin deficiency (Gómez-Mariano et al. 2020). Furthermore, combining organoids with gene editing tools such as the CRISPR/Cas 9 system has opened up a wealth of opportunity, as organoids are highly amendable to genome editing (Fujii et al. 2019). For instance, the application of organoid models in tumour-biology studies has provided a patient-specific functional testing platform for drug administration and sensitivity, as both phenotypic and genomic results can be retrieved, molecular mechanisms can be identified and personalized treatment strategies can be initiated (Clevers 2016; Drost and Clevers 2018; Huch et al. 2017). These pioneering works would potentially allow the identification of biomarkers and further personalized treatments (Huch et al. 2017).

## Conclusion

Single-cell genomic approaches are changing the concept of personalized medicine from early detection to tailored treatments. The harmonization and standardization of single-cell technologies is leading to translational applications from bench to bed. The identification of new and rare cell types in an early stage, the precise monitoring of their molecular changes and their contribution to disease pathogenesis and outcome are key stepping stones for implementing these technologies in the clinical practice.

Still future efforts will be needed to dissect how complex diseases are influenced by lifestyle, dietary interventions and ageing. Single-cell multiomics is emerging as a novel technology to read out multiple layers of genetic and epigenetic information simultaneously, aiming to anticipate changes in cell fate and cellular function. Overall, single-cell genomics is opening a new frontier in the field of personalized medicine leading to the next generation of health care.
